# Role of Inflammation, Oxidative Stress, and Mitochondrial Changes in Premenstrual Psychosomatic Behavioral Symptoms with Anti-Inflammatory, Antioxidant Herbs, and Nutritional Supplements

**DOI:** 10.1155/2022/3599246

**Published:** 2022-07-13

**Authors:** Arshiya Sultana, Khaleequr Rahman, Md Belal Bin Heyat, Sumbul, Faijan Akhtar, Abdullah Y. Muaad

**Affiliations:** ^1^Department of Amraze Niswan wa Ilmul Qabalat, National Institute of Unani Medicine, Ministry of AYUSH, Bengaluru, Karnataka, India; ^2^Department of Ilmul Saidla, National Institute of Unani Medicine, Ministry of Ayush, Bengaluru, Karnataka, India; ^3^IoT Research Center, College of Computer Science and Software Engineering, Shenzhen University, Shenzhen, Guangdong 518060, China; ^4^School of Computer Science and Engineering, University of Electronic Science and Technology of China, Chengdu, Sichuan, China; ^5^IT Department, Sana'a Community College, Sana'a5695, Yemen

## Abstract

Premenstrual syndrome (PMS) significantly lowers the quality of life and impairs personal and social relationships in reproductive-age women. Some recommendations are that inappropriate oxidative stress and inflammatory response are involved in PMS. Various nutritional supplements and herbs showed neuro-psycho-pharmacological activity with antioxidant and anti-inflammatory properties. This study aims to determine the systematic review of randomized controlled trials (RCTs) of herbal medicine and nutritional supplements in PMS. We also comprehensively highlighted the role of oxidative stress, inflammation, and mitochondrial changes on PMS with the application of computational intelligence. We used PRISMA and research question-based techniques to collect the data for evaluation of our study on different databases such as Scopus, PubMed, and PROSPERO from 1990 to 2022. The methodological quality of the published study was assessed by the modified Jadad scale. In addition, we used network visualization and word cloud techniques to find the closest terms of the study based on previous publications. While we also used computational intelligence techniques to give the idea for the classification of experimental data from PMS. We found 25 randomized controlled studies with 1949 participants (mean ± SD: 77.96 ± 22.753) using the PRISMA technique, and all were high-quality studies. We also extracted the closest terms related to our study using network visualization techniques. This work has revealed the future direction and research gap on the role of oxidative stress and inflammation in PMS. In vitro and in vivo studies showed that bioactive molecules such as curcumin, allicin, anethole, thymoquinone, cyanidin 3-glucoside, gamma-linoleic acid, and various molecules not only have antioxidant and anti-inflammatory properties but also other various activities such as GABA-A receptor agonist, serotonergic, antidepressant, sedative, and analgesic. Traditional Unani Herbal medicine and nutritional supplements can effectively relieve PMS symptoms as they possess many bioactive molecules that are pharmacologically proven for the aforementioned properties. Hence, these biomolecules might influence a complex physical and psychological disease process like PMS. However, more rigorous research studies are recommended for in-depth knowledge of the efficacy of bioactive molecules on premenstrual syndrome in clinical trials.

## 1. Introduction

Premenstrual syndrome (PMS) is categorized by psychosomatic and behavioral symptoms that manifest repetitively in the luteal phase, a cyclic pattern, and days before menstruation. The patient is symptom-free between two luteal steps [1–3]. The global pooled prevalence of PMS is 47.8% with the highest in Asia and the lowest in Europe [4]. Eighty-five per cent of reproductive-age women experience at least one symptom of PMS, and 2.5–3% of women suffer from premenstrual dysphoric disorder (PMDD) [2]. It has been reported that women with PMS tend to have a significantly lower quality of life, legal problems, suicidal ideation, decreased work productivity, social isolation, parenting problems, increased absenteeism from work, impaired personal and social relationships, and more frequent visits to hospitals [2, 5, 6]. Additionally, PMS also leads to an increased tendency to have an accident, drug addiction, economic losses, and a decline in demic achievement. The diagnostic criteria of the PMS for affective symptoms and somatic symptoms must be experienced during the five days before menses in three prior menstrual cycles and relieved with the menstrual flow [7]. The primary psychological range of PMS is anxiety and depression [6, 8].

Sex hormones strongly modulate the immune-inflammatory process [9]. PMS causes are still unclear and are multifactorial [10, 11] probably as a consequence of interaction and biochemical changes amid the menstrual cycle's hormonal fluctuations (imbalance like progesterone deficiency and estrogen excess) [8] with central neurotransmitters (*γ*-aminobutyric (GABA) and cholecystokinin serotonin) and altered brain processes [5], genetic vulnerability, and regulation of the renin-angiotensin-aldosterone system [12–14]. Previous research reported that various other factors such as high carbohydrates, fat, lack of calcium, vitamins and minerals, psychological factors (stress, depression, and anxiety), genetics, and lifestyle (alcohol consumption, smoking, lack of exercise, and eating habits) in addition to oxidative stress and inflammation are related with the incidence of PMS. Oxidative stress is higher in women who experience PMS as per the previous research [5]. Consequently, as an etiology is unclear, several possible treatments are prescribed and purchased [8]. Various therapeutic interventions have been scientifically confirmed helpful in PMS ranging from nonpharmacological management (cognitive behavioral therapy, lifestyle modification and diet, education, exercise, and complementary and herbal medications) to pharmacotherapy hormonal therapy (gonadotropin-releasing hormone (GnRH) agonists OCP, etc.) and psychotropic medications (benzodiazepines and selective serotonin reuptake inhibitors) [13]. Despite a reasonable success rate of these pharmacotherapies for PMS, each has a substantial adverse effects profile (insomnia, dysphoria, nausea, perspiration, muscle cramps, and tremor). Women with PMS symptoms are frequently hesitant to take SSRIs and other conventional medicines, partially because it causes side effects. Thus, the majority of women are turning towards complementary and alternative medicines to treat their symptoms. Many Unani herbs have been anticipated to alleviate PMS as they are a potentially effective natural alternative for PMS symptoms [8].

In recent times, products from plants and animals that are natural sources appear to be a potential basis of emergent pharmaceutical agents and food supplements [15]. The natural products or foods have active compounds that are also established to have antioxidant property and are replacing synthetic antioxidants and antimicrobial agents due to their carcinogenicity [16]. Evidence from recent research confirmed that vitamins and supplementation (magnesium, calcium, vitamins B6, D, and E) or herbal/complementary and alternative medicine (CAM) might alter oxidative stress and the hormonal or inflammatory profile in women who experience PMS. CAMS including supplements and vitamins are preferred by women affected by PMS over conventional medical or surgical management to help cope with symptoms. It has been implicated that oxidative stress and antioxidants may affect a complex psychological and physical disease process like PMS [1]. Various Unani herbs such as polypody, saffron, chaste berry, anise seed, black seeds, spikenard, lemon balm, borage, chamomile, ginger, fennel, and serely have been studied in premenstrual syndrome. To avert the beginning and development of neurodegeneration by amendment of pathogenic factors in neurodegenerative disorders, phytochemicals can unswervingly inhibit or promote mitochondrial apoptosis cascade and regulate mitochondrial functions, ROS*/*RNS production, apoptosis signaling, mitochondrial biogenesis ATP synthesis, and degradation by autophagy (mitophagy) and exhibit neuroprotection. Relatively, the vast number of compounds, including anti-inflammatory, antioxidants (vitamins C and E), inhibitors of monoamine oxidase (MAO), and coenzyme Q (bioenergetic compound agents), has been testified to be neuroprotective [17]. The “mitochondrial matrix thiol system” has a vital role in antioxidant defense [18]. In addition, bioactive food compounds of herbs and spices show both antioxidant and anti-inflammatory processes [19]. Flavonoids show anti-inflammation property through the antioxidant property and inflection of signal transduction for the amalgamation of pro-inflammatory cytokines [17].

Previously, Dante and Facchinetti [20] designed a review on herbal medicine related to PMS with 10 RCTs. They also designed a study related to herbal medicine and acupuncture for PMS in 2014 [21]. The other researchers conducted a study on the Chamomile single herb [8] and exercises for PMS [22]. However, a recent update on the systematic review of RCTs on herbal medicine and nutritional supplements alleviating PMS as per the modified Jadad scale was not available to the best of our knowledge. Hence, we aimed and intended to reconnoitre the critical appraisal and contemporary research to address the updated systematic review of RCTs as per the modified Jadad scale to provide more objective data for the effectiveness of herbal medicine and nutritional supplements in alleviating PMS symptoms.

In addition, computational intelligence has an important role in the medical areas such as detection, treatment, localization, and recommendation. Currently, some intelligence techniques such as machine learning, deep learning [23, 24], and the Internet of Things (IoT) [25] with processing techniques such as signal processing, image processing, text processing, video processing, and audio processing are used in the medical fields. Previously, the Siddiqui group used physiological signals to detect sleep disorders [26–29]. Lai group used medical machine learning and deep learning techniques to automatic detection of bruxism sleep disorder [30–32] and cardiac diseases [33, 34]. Ali et al. [35] designed an automatic system for the detection of Parkinson's disease. Ukwuoma et al. [36] used different deep learning technique to classify and detect medical diseases. The Iqbal group applied intelligence techniques for the analysis of the experimental data [37, 38]. However, in this present study, we used the computational intelligence technique to classify the PMS experimental data.

We accumulated up-to-date research to address oxidative stress, inflammation, and mitochondrial changes and their associates. We conducted this review to address most of the information related to PMS with oxidative stress, inflammation, and mitochondrial changes. In addition, we also gave the idea about using computational techniques on PMS. Because no previous research has incorporated this type of information in the review article, this review aims to overview and analyze various issues related to inflammation, oxidative stress, and mitochondrial changes in premenstrual psychosomatic and its behavioral symptoms towards the therapeutic implication of herbal anti-inflammatory and antioxidants. Hence, this paper aims to provide a solution to the following research questions (RQs):

- What is the etiopathogenesis, the role of oxidative stress, inflammation leading to mitochondrial changes in premenstrual psychosomatic, and its behavioral symptoms?
- What are the various mechanisms of action of nutritional supplements and herbal medicines with their phytochemical/bioactive constituents in premenstrual psychosomatic and its behavioral symptoms?
- Does computational intelligence have a role in PMS data analysis for future modulation of premenstrual symptoms?

We designed a comprehensive picture related to the role of inflammation and oxidative stress during symptoms based on the previous research with the recent advancement and future recommendations. We used two types of literature review: (A) systematic literature review using PRISMA guidelines on Scopus, PubMed, and PROSPERO databases [39] and (B) research questions based literature review [39, 40]. Our main contributions to this study are as follows: (a) to design a comprehensive survey on the effectiveness of herbal medicine in PMS symptoms, (b) the role of oxidative stress and inflammation on PMS symptoms, (c) to find various mechanisms of action of nutritional supplements and herbal medicine, (d) computational intelligence-based classification of the experimental data using support vector machine (SVM) and random forest model, (e) to design a network visualization and word cloud based on previously published articles, and (f) to determine the research gaps and future directions. The present study's organization is as follows: a systematic literature review using PRISMA [41] and an answer to the RQs [39, 40] based on previous studies, network visualization [42, 43], and world clouds [44] based on keywords, discussion, research gap, future prospects, and conclusions.

## 2. Systematic Literature Review Based on PRISMA

### 2.1. Method

A comprehensive research methodology is systematically discussed, together with various data collection and analysis steps. PRISMA guidelines [39–41, 44, 45] were implemented to produce this study as it uses a guideline checklist [46]. Various steps carried out in the methods are as follows: (a) planning and developing a protocol; (b) conducting a comprehensive literature search; (c) data collection by screening titles, abstracts, and keywords; (d) developing explicit selection criteria; (e) analyzing the results; (f) synthesizing the information; and (g) reporting results [47, 48]. Additionally, a classification method for computational intelligence in an experimental study was also performed.

#### 2.1.1. Database and Search Strategies

We retrieved online databases such as PROSPERO, PubMed, and Scopus to collect all literature from 1990 to 2022 focusing on oxidative stress, inflammation of psychosomatic and behavioral premenstrual symptoms among reproductive-age women, the therapeutic implication of antioxidants, and anti-inflammatory nutritional supplements and herbal medicines. Additionally, a classification method for computational intelligence in an experimental study was performed. To get an adequate number of studies related to our topic, we broadened MeSH search terms and categories. The keywords used for the literature search were as follows: “Premenstrual Syndrome”, “Premenstrual Dysphoric Disorders”, “Herbal medicine and the premenstrual syndrome”, “oxidative stress and PMS”, “inflammation and PMS”, “psychological symptoms and the premenstrual syndrome”, “antioxidants and the premenstrual syndrome”, and “anti-inflammatory herbal medicine and PMS”.

#### 2.1.2. Eligibility Criteria

The selection of articles was performed in two steps. In the initial step, one of the authors screened the articles based on the titles and abstracts. Two researchers independently analyzed and extracted the data from the screened articles after reviewing them thoroughly to avoid bias, and the results were organized. Detailed records regarding age, tools for data collection, intervention type, significant results, phytoconstituents, and pharmacological activities were recorded. Irrelevant studies and insufficient quantitative data were excluded. The PRISMA and Consort Statement Checklist were used for assessing reliability, while duplicates of the studies were recognized and deleted.  Table 1 summarizes the inclusion and exclusion criteria considered based on various parameters. Research articles from peer-reviewed journals and dissertations were included, and those not directly related to our framework of the study were excluded. Data from animal experiments and *in vitro* investigations were included for understanding the etiopathogenesis of premenstrual symptoms and pharmacological activities of herbs. In the final comparison, studies were considered reliable and were included, if they had standard criteria. All data retrieved were read and assessed by researchers and consecutively checked individually by the other authors.

#### 2.1.3. Study and Process of Data Extraction

We studied the titles, abstracts, and keywords of all published articles to determine their eligibility and relevance to be included in our study. After a thorough examination of the full articles, final decisions on inclusion were made. To exclude duplicated publications, the complete versions and most recent articles were selected.

**Figure 1:**
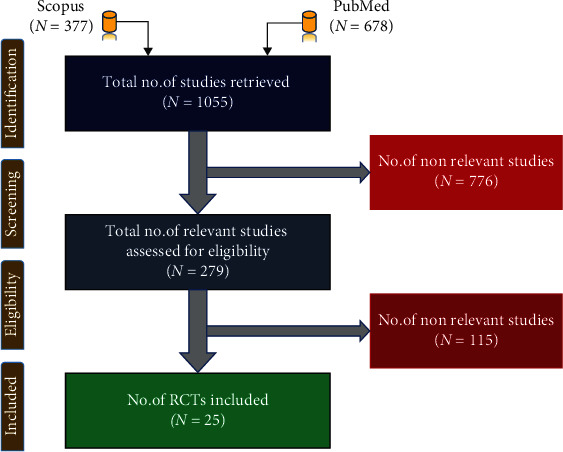
PRISMA of the proposed study.

Figure 1 depicts the total articles retrieved from PubMed and Scopus databases. Finally, we included 25 RCTs for systematic analysis and irrelevant articles were excluded. We included one recent dissertation work and a few articles from Google Scholar. Research articles written in Chinese language were excluded. Next, these selected articles were further screened by abstracts, titles, and keywords, considering the exclusion criteria.  Table 1 shows the various inclusion criteria.

#### 2.1.4. Risk of Bias and Quality Assessment

The risk of bias and the quality of published articles included were assessed by 2 researchers (AS and S). Any kind of discrepancy was resolved by consensus among the authors. Data was successfully extracted and independently assessed for the quality assessment of the collected data from primary studies. We evaluated the methodological quality using a modified Jadad scale which includes 1 point for each domain described with a minimum score of zero and a maximum score of 8 points. The higher scores indicate better high quality (>3) and <3 signified low quality [49, 50].

### 2.2. Analysis and Synthesis of the Results

**Figure 2:**
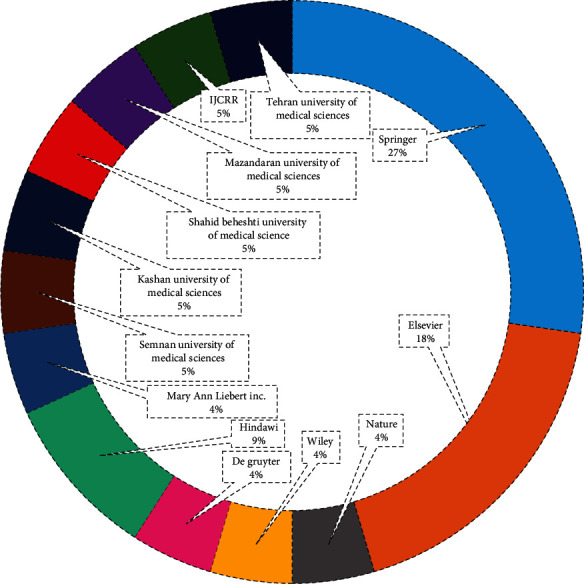
Publisher-wise previously published articles related to RCTs on PMS and herbal medicine.

**Figure 3:**
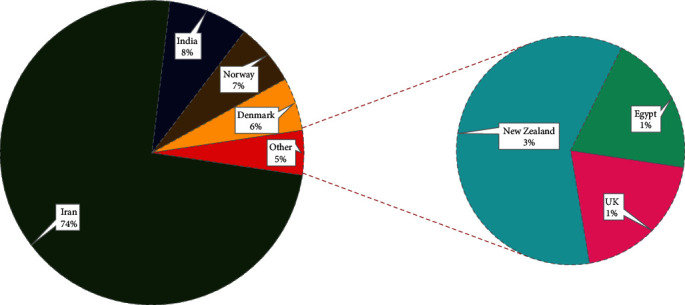
Country-wise previously published articles related to RCTs on PMS and herbal medicine.

**Figure 4:**
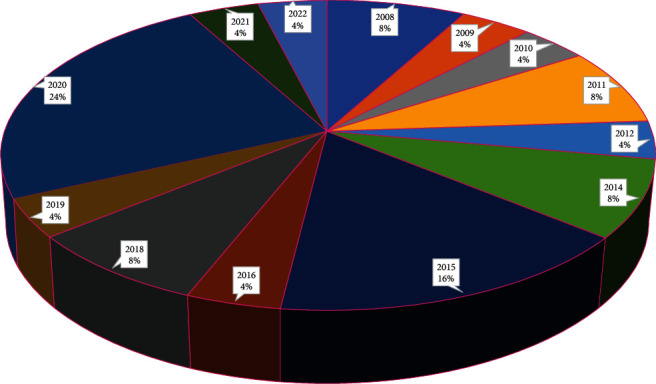
Year-wise publications related to RCTs on PMS and herbal medicine.

We used online databases, Scopus, PubMed, and PROSPERO to collect the data. All published papers were written in different languages except English and Persian in Scopus and PubMed, so we did not add those studies to our work. Few articles were included from the dissertation work and other databases. We applied “Premenstrual” AND “Herbal Medicine” “Premenstrual” AND “oxidative stress and inflammation” to the Scopus database and collected all information about the papers. We found subject areas, year-wise publications, country-wise, and sources shared in the publications related to the premenstrual symptoms among reproductive-age women mentioned in Figures 2, 3, and 4.  Figure 2 represents the publisher-wise publications of RCTs published on premenstrual syndrome and herbal medicine. The majority of papers were published by Springer (27%), followed by Hindawi (9%) and other publishers. Besides, Figure 3 represents the country-wise publications based on previous reports. The Iran researchers have a maximum of 74% contribution in the publications, followed by India (8%), Norway (7%), Denmark (6%), and others (5%).  Figure 4 represents the yearly publications published from 1990 to 2022. Leading publications were found in 2020 (24%) followed by 2015 (16%). Three papers were in the Persian language, and the others were from the English language.

In Table 2, the characteristics of previously published papers using PRISMA techniques were evaluated through design, number of participants, tools, intervention type, outcomes of the study, and adverse effects of nutritional supplements and herbal medicine. Additionally, bioactive molecules and pharmacological activities were also included in the same table for ease to understand the effect of herbal medicine and nutritional supplements. PRISMA techniques only covered the given keywords on the Scopus and PubMed databases for RCT studies. We also included one dissertation work. We did not cover the other indexing, so our research questions covered most previously published articles. We found 25 randomized controlled studies with 1949 participants (mean ± SD: 77.96 ± 22.753; variance: 517.71; and CI: 4.55) using the PRISMA technique. We found four RCTs, which were on nutritional supplements, and 21 RCTs where herbal medicines were used for PMS symptoms. All the studies were randomized, five were single-blind, three were triple-blind, and 17 studies were double-blind. In 25 studies, 15 studies reported the adverse effects. Most of the studies interpreted the action and pharmacological properties of herbal medicines.

#### 2.2.1. Risk Assessment of the Data

In the 25 included studies, the risk of bias in each of the 8 domains is shown in Table 3. Most of the studies indicated a substantial quality of this systematic review with low risk for all domains. The quality of included studies was high in that 6 studies had scores of 8 and 19 studies had scores between 5 and 7 on the modified Jadad scale.

**Figure 5:**
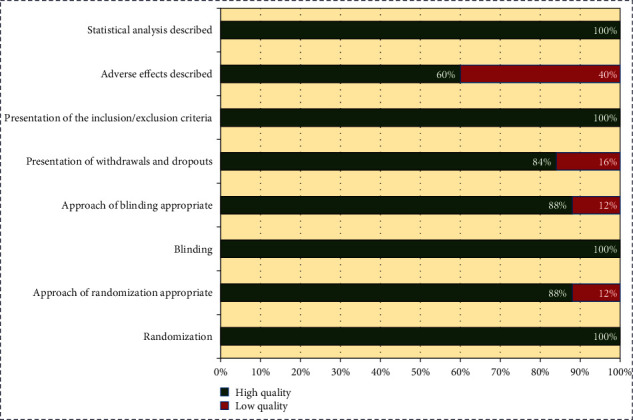
Quality assessment of randomized controlled trials.

The systematic literature review of 25 RCTs was having low risk of bias with high quality as per the modified Jadad scale. 100 percent of studies were randomized and mentioned selection criteria, blinding process, and statistical analysis. 88% of studies reported the appropriate method of randomization, and 60% of studies have not reported the adverse effect of the drugs (see Figure 5).

## 3. Literature Review Based on Research Questions

Table 2 covered RCTs on nutritional supplements and herbal medicines used to alleviate premenstrual syndrome among reproductive-age women. Further, we designed research questions related to premenstrual psychosomatic and behavioral symptoms and oxidative stress, inflammation, and various mechanisms of action in nutritional supplements and Unani herbal medicines with their phytochemical constituents/bioactive molecules in premenstrual psychosomatic and its behavioral symptoms. Further, for the accuracy of data, computational intelligence has a role in RCT data analysis for future modulation of premenstrual symptoms and in designing a network visualization and word cloud based on previously published articles. Hence, the research questions were answered.

### 3.1. What Is the Etiopathogenesis, the Role of Oxidative Stress, Inflammation Leading to Mitochondrial Changes in Premenstrual Psychosomatic, and Its Behavioral Symptoms?

PMS has a variety of stress symptoms together with psychiatric and somatic complaints, and oxidative imbalance appears to be implicated in the biochemical basis of the pathophysiologic mechanism of PMS [14]. PMS causes are still unclear and are multifactorial [10, 11]. Currently, few theories propose that there is a relationship between ovarian hormone levels and PMS. PMS is related to ovarian hormone levels based on the fact that the absence of PMS symptoms before puberty, during pregnancy, after menopause, and during treatment with gonadotropin-releasing hormone (GnRH) analogue conditions. Nevertheless, it has been proven that there is no noteworthy difference between symptomatic and asymptomatic women concerning progesterone levels. This is explicated by the concept that some women are more sensitive to progesterone. Additionally, serotonin levels and gamma-aminobutyric acid (GABA) levels also play a role in premenstrual symptoms. Ovarian hormones, estrogen, and progesterone are influenced by erotogenic activity in the brain and are sensitive to serotonin receptors. Progesterone causes depression in an individual by increasing MOA, related to the transport of serotonin; however, estrogen produces an antidepressant effect. Consequently, high progesterone and low estrogen levels in the luteal phase trigger depressive mood. Another concept for the etiology of PMS is the relationship between GABA and progesterone. Allopregnanolone, a metabolite of progesterone, regulates the level of GABA in the blood. Before the luteal phase, allopregnanolone is in higher concentration; therefore, GABA receptors are less susceptible to allopregnanolone. Consequently, allopregnanolone's lower concentration in the luteal phase causes aggression, depression, and mood anxiety. Certainly, women suffering from PMS showed low levels of allopregnanolone levels [6].

PMS patients showed a lower 5-HT response to a 5-HT precursor, tryptophan, during the luteal phase when compared to the follicular or mid luteal phase. Currently, another possible mechanism under search is an insufficient inflammatory response to biological or physical stimuli and the manifestation of oxidative stress “an imbalance between the production of reactive oxygen species (ROS) and their inactivation by antioxidant protection mechanisms.” In a healthy human, as a result of metabolic activity, not only inflammation but also oxidative stress can be seen. Sometimes, inflammation supports cells to accustom to and subsist stress conditions. During the menstrual cycle, alternation of inflammation status is experienced by reproductive-age women. Nevertheless, oxidative stress and chronic inflammation are considered probable factors of PMS development, pre-eclampsia, endometriosis, and recurrent pregnancy loss. Biomarkers of oxidative stress and inflammation were measured in women with PMS symptoms. Another principal theory is an alternation in response to exposure or withdrawal to the progesterone metabolite, allopregnanolone, which is also a gamma-aminobutyric acid (GABA) agonist. It has been proven that premenstrual symptoms are reduced by blocking allopregnanolone production, and a serotonin re-uptake inhibitor is one of the PMS pharmacotherapies that affects allopregnanolone levels [10].

Mitochondria have a key role in cellular bioenergetics and cell survival. Oxidative stress results in chronic hypoperfusion and induces mitochondrial damage. The increased intracellular production of oxidants and pro-oxidants is associated with mitochondrial damage [74]. Mitochondrial dysfunction possibly will initiate downstream variations in extracellular matrix proteins (EMP) such as inflammation, neuronal nitric oxide (nNOS), reelin, and finally adult hippocampal neurogenesis in oxidative stress. The reelin and mitochondrial dysfunction relationship has been studied where reelin in the periphery cooperates with the immune system; hence, a loss of reelin can amplify inflammatory markers that affect mitochondria. Additionally, some studies reported that variations in mitochondrial functions such as membrane polarity and oxidative phosphorylation, potentially increasing apoptosis, oxidative, and stress, may precede the development of depressive symptoms. Serotonin (5HT) and norepinephrine (NE) receptor activation and desensitized and dysregulated monoamine receptors result in deviations in intracellular second messenger signal transduction cascades associated with depression. These explanations are linked to mitochondrial dysfunction where the binding of neurotransmitters to receptors is required for the activation of downstream signaling of ATP. Moreover, patients with mitochondrial DNA (mtDNA) mutations or mitochondrial diseases frequently present symptoms distinctive from mood disorders. Remarkably, oxidized mtDNA triggers pro-inflammatory cytokines and increases inflammation and plays a significant role in the progression of depressive symptoms. Cognitive dysfunctions are a common symptom related to depression, and studies reported that cognitive impairments in mice and humans are caused by variations in mtDNA. The association between mitochondrial dysfunction and psychopathology has been evidenced by a correlation found between depression rating scale scores and altered biochemistry. Indeed, depression, anxiety, autism, psychotic disorders, Alzheimer's disease, and Huntington's disease are connected to ATP reduction and its relation to oxidative stress [18, 75]. Hence, the expression of NOS enzymes is excited by hypoperfusion in the brain tissues caused by prolonged oxidative stress, which accelerates the development of ROS and RNS. ROS and RNS together contribute to the blood-brain barrier (BBB) dysfunction and brain parenchymal cell damage [74]. Studies showed that small increases in the immune mediator C4 and lowered antioxidant defenses (PON1 and AREase) are related to an increase in premenstrual symptoms during the menstrual cycle. Moreover, estradiol and progesterone possibly have a protective role to counter oxidative stress [76].

The common features of PMS are psychiatric (depression, anxiety, etc.) and somatic disorders that involve the etiology of chronic inflammation, which ensues when cytokine-producing cells remain activated. A prevalence study on PMS women reported a correlation between inflammatory factors elevated levels (IL-2, IL-4, IL-10, and IL-12) and the total symptom score in PMS, and further, higher correlations were noted for IL-12, IL-10, and PMS symptoms [14]. P21-activated kinase 1 (PAK1) plays a critical role and contributes to various diseases including inflammation, immunosuppression, cancer, viral infection, ageing, and diabetes [77]. Zeinolabediny et al. [78] discussed the inflammatory disease associated with cardiovascular complications. TNF-*α*, CRP, and IL-6, the inflammatory markers, are related to migraine headache, a usual symptom of PMS. Johnson et al. [79] reported that there was the strongest correlation between IL-10 and IL-12 levels among inflammatory indices and the total score of symptoms in PMS women. One of the studies showed the relationship between anxiety and IL-10 and a positive relationship in female soccer players with PMS post-game in the luteal phase [80].

### 3.2. What Are the Various Mechanisms of Action in Nutritional Supplements and Herbal Medicines with their Bioactive Molecules in Premenstrual Psychosomatic and Behavioral Symptoms?

As per the suggestions, PMS management needs a progressively multidisciplinary team that has embraced an integrated holistic method. Similarly, a personalized therapy and management plan for an individual would be practical as the type, number, and severity of PMS symptoms vary from individual to individual. For example, if premenstrual symptoms are mild to moderate, diet and lifestyle changes cause a cure. Pharmacological treatment is suggested if the symptoms start to have an adversarial effect on daily life. The initial step in PMS management is to make awareness regarding education and consultancy, self-care, and self-screening practices. Next, treatment includes nonpharmacological and pharmacological approaches. Lastly, the option is surgical methods.

Many plant products are useful for the alleviation of PMS symptoms. In a RCT, *Polypodium vulgare* showed a significant reduction in the premenstrual symptoms scale (PMSS) and HRQoL [58]. *Polypodium vulgare* Linn.(*Bisfayej*) is indicated for various ailments such as melancholia, epilepsy, dementia, arthritis [81, 82], abdominal pain, and other neurological diseases as it possesses brain and heart exhilarant properties [82]. Herbal medicines are pharmacologically proven for various properties such as tranquillizing effect, memory retention improvement, analgesic, aldosterone antagonists, immune-modulator, antioxidant, anti-inflammatory, cholinesterase inhibitory activity, smooth muscle relaxant, CNS depressant, and dopamine effect [83–86] (see Table 2). *P. vulgare*, *C. sativus*, *Vitex agnus castus*, anise seed, *Nigella sativa*, *N. Jatamansi*, *M. officinalis*, *Echium amoenum*, chamomile, *H. perforatum*, *G. biloba*, *Z. officinalis*, fennel, and serelys consist of various inorganic constituents and organic constituents (flavonoids, resins, tannins, steroids, and protein) (see Table 2) that have antioxidant and anti-inflammatory properties. Flavonoids exhibit anti-inflammation activity through antioxidant property and inflection of signal transduction for the pro-inflammatory cytokines synthesis [17]. Randomized controlled studies carried out with herbal medicines and nutritional supplements to treat the symptoms of PMS are summarized in Table 2. The placebo group in this study also had amelioration in the severity of somatic and psychological symptoms that probably accredited to the psychological effect of the placebo. In various studies, the significant response to placebo and the usefulness of herbal medicines in PMS management was pragmatic as a usual finding. This might be clarified by the circumstances that using beneficial methods give women a sensation of augmented self-control over their lives. Hence, minor interventions like placebo perhaps lead to required effects [13]. An observational analytical study on 90 students aimed to determine the association between mental status, levels of 8-OhdG, and nutritional intake in urine with PMS events [5].

**Figure 6:**
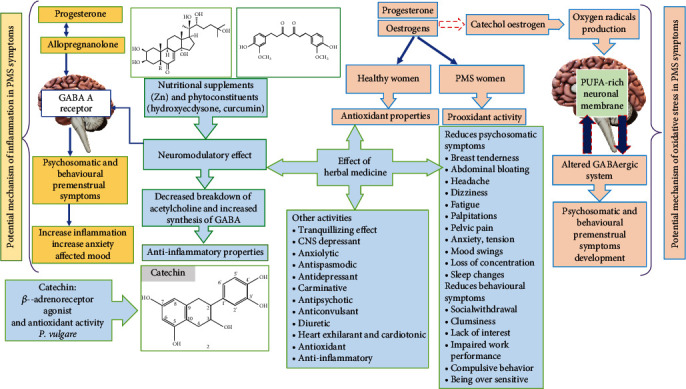
Potential mechanisms of inflammation and oxidative stress in PMS. Antioxidant and anti-inflammatory effects of nutritional supplements and herbal medicines.

PMS symptoms are linked to the increased inflammatory reactions and oxidative stress as well as super activation of the renin-angiotensin-aldosterone system [87]. The literature demonstrates the relationship between diverse symptoms connected to the menstrual cycle and a multitude of other psychological and physical diseases and sustenance for the practice of antioxidants in managing them. Nevertheless, the precise mechanisms are unclear; we theorized that oxidative stress and antioxidants might also probably affect a complex psychological and physical disease process such as PMS. A research [1] described that women with PMS had higher concentrations of oxidative stress markers. The positive relations between F2-isoprostane with psychological symptoms (anger, tension, and crying) may perhaps be explicated by an original disturbance in the neurotransmitter GABAergic neuroendocrine system by reactive oxygen species (ROS). ROS causes variations in these neurotransmitter levels and may probably cause neuronal cell damage, producing behavior changes (increased appetite and obesity) and mood and the progress of depression and anxiety symptoms [1]. Estrogen and progesterone in healthy women act as antioxidants, whereas in PMS women the inappropriate increase in pro-oxidant activity may lead to oxidative damage to the polyunsaturated fatty acid-rich neuronal membrane (Figure 6). Consequently, dysfunction of the GABAergic system perhaps leads to the PMS symptom development. Catechol estrogens, which produce oxygen radicals, are an additional cause of oxidative damage occurring in the neuronal membrane [10]. A protective role of estradiol in mitochondria has been reported in some studies. It possibly inhibits the ROS passage in the mitochondria and increases the rate of ATP synthesis and prevents mitochondrial collapse. Mitochondrial functions such as ROS production and apoptosis are affected by the biosynthesis of OXPHOS enzymes. It appears that mitochondrial estrogen and glucocorticoid receptors (GRs) in lung tissue are intricated in oxidative stress and mitochondria are receptive to changing levels of estradiol and stress hormones. Dysfunctional mitochondria cause a negative cascade that might eventually increase inflammatory responses, oxidative stress, and proapoptotic events; a few of them intricated in the pathogenesis of depression [18].

The antioxidant is currently any chemical that prevents, slows, or reverses oxidative damage in the target molecule [81]. It is widely known that flavonoids have a higher antioxidant capacity due to their capacity to scavenge free radicals [81]. Flavonoids are direct scavengers of ROS and RNS downregulation of radical-producing ones and upregulation of ROS-removing enzymes, hence acting as strong antioxidants. Flavonoids produce a hydrogen molecule, a phenoxy radical, which scavenges single oxygen, hydroxyl, peroxyl, and superoxide radicals by releasing another hydrogen. The diol group suppresses ROS generation by forming a composite with copper, ferric iron, and other transition metal ions. Superoxide dismutase, glutathione S-transferases, catalase, glutathione reductase, glutathione peroxidase, and NAD (P)H: quinone oxidoreductase (NQO1) are all upregulated by polyphenols. “The production of inflammatory signal molecule peroxiredoxin 2 (PRDX2) and the activation of macrophages to generate IL-6 are both important roles for ROS in inflammation.” Flavonoids inhibited the synthesis and release of inducible nitric oxide synthase, inflammatory IL-6, TNF-a, and MCP-1 by inhibiting NF-*κ*B, AP-1, and other pro-inflammatory transcription factors. Through the second messenger modulation (cGMP, cAMP, calcium, and protein kinases), flavonoids suppress the formation of inflammation mediators such as prostaglandins, leukotrienes, and arachidonic acid and inhibit the activity of COX and lipoxygenase and arachidonic acid-metabolizing enzymes [17].

Polypody rhizome contains various organic compounds like resins, tannins, steroids, flavonoids, glycosides, proteins, and reducing sugars and various inorganic compounds like iron, calcium, magnesium, potassium, sulphur, and chloride. Its ash contains a large amount of carbonate of potassium. Polypody rhizome contains active constituents phytoecdysteroids, identified as “20-hydroxyecdysone and polypodine B.” Numerous researches reported the antioxidant and neuroprotective effects of 20-hydroxyecdysone. Polypody rhizome contains flavonoids that have been proven for their antioxidant property [85]. They probably act as antioxidants to inhibit lipid peroxidation and free-radical-mediated cytotoxicity, or as weak estrogenic agonists or antagonists to modulate endogenous hormone activity. They contain conjugated ring structures and hydroxyl groups which might probably function as antioxidants, *in vitro*, or in cell-free systems by scavenging superoxide anions, chelating redox-active metals, and singlet oxygen or complexing with oxidizing species. The contents of flavonoids in the extracts of *Rhizome polypodii* as per HPLC-DAD are catechin, naringenin, resveratrol, and quercetin. Low concentrations of quercetin scavenged free radicals, however, inhibiting oxidative DNA breakage [88]. Another study result demonstrated that the methanolic extract of *P. vulgare* contains significant amounts of flavonoids such as epicatechin, catechin, shikimic acid, and caffeoylquinic acid derivatives showing cellular repair and cytoprotective activities. The high content of polyphenolic compounds may be responsible for this biological activity [88]. Monoamine oxidase (MAO) functions as a modulator of signal pathways involved in neuronal survival and death. It is found on the outer membrane of mitochondria and helps to maintain monoamine neurotransmitter homeostasis in the brain. Antidepressants are made up of MAO inhibitors of type A (MAO-A). MAO is inhibited by flavonoids like catechin and quercetin. Quercetin, a flavonoid, inhibits MAO-A. “Flavonoids activated upstream MAPK–kinase–kinase, inhibited oxidative stress-induced apoptosis, and prevented Jun N-terminal kinase activation.” Catechin raises serum brain-derived neurotrophic factor in humans (BDNF). Tropomycin-related kinase B (TrkB) and tropomycin-related kinase A (TrkA) expression and neurogenesis are increased by polyphenols, resulting in neuroprotection and antidepressant activity [17]. Tannins may work as antioxidants to scavenge free radicals and stop such damaging reactions [85]. It is advocated that mitochondrial energy metabolism may perhaps have an antidepressant mechanism of action [18]. Moreover, the bioflavonoids present in *P. vulgare* are primarily known as stress modulators. It is recommended that the serotonergic mechanism is involved in the antidepressant effect [72]. *P. vulgare* is one of the constituents of *Sharbat-e-Ahmed Shah*, a compound formulation useful in depression and insomnia in traditional medicine since antiquity as an antidepressant activity [89]. Researchers reported that this compound was able to increase the availability of tryptophan, a 5HT precursor in the blood and brain, and hence increase 5-hydroxytryptamine (serotonin: 5HT) in the brain, thus causing anxiolytic and antidepressant activities in experimental rats [89]. In our study, polypody was able to reduce the psychological symptoms as it has antidepressant properties and increases the availability of tryptophan as studies have proved that PMS patients show a lower 5-HT response to tryptophan during the luteal phase. An animal study in anaesthetized dogs that showed *P. vulgare* extract showed hypotensive activity due to *β*-adrenoreceptor agonist activity and vasodilation effect that was caused due to phytochemical catechins [83, 89].

It is hypothesized that sex steroids and allopregnanolone (a derivative of progesterone) may influence the PMS symptoms as their receptors are prevailing in the hypothalamus or amygdala of the brain and they easily pass the blood-brain barrier. Allopregnanolone (a progesterone metabolite) is an agonist of GABA A and depending upon its concentration has different actions. For example, it has anxiolytic and sedative effects at high concentration, whereas it may cause depression and negative mood at a lower concentration. As in the lacteal phase, GABA A receptors turn out to be less sensitive to allopregnanolone after exposure to its high concentrations, resulting in increased premenstrual symptoms. The relation between inflammation and allopregnanolone is relatively complex: Animal study results specify that “agonists of GABA A receptors attenuate the impact of inflammation, while the inhibition of GABA A receptor activity increases proinflammatory effects.” Allopregnanolone decreases anxiety and improves mood (Figure 6) [10].

Typical ecdysteroids present in *P. vulgare* are “20-hydroxyecdysone (20E) and polypodine B (polB).” A research report showed that phytoecdysteroids present in P. *vulgare* probably impact the activity of the CNS not only because of neuromodulatory effects on the GABA A receptor as well as partly due to neurotransmitter metabolism (decreased breakdown of acetylcholine and increased synthesis of GABA) and enhanced activity of the immunologic system, antioxidant, antimicrobial, and antiproliferative properties [90]. Furthermore, the rhizome of *P. vulgare* L. has been found to possess a protective effect in drug-induced catalepsy, thus suggesting that it enhances the transmission of dopamine in the CNS and has been explored for various psycho-neurological disorders. The rhizome extract possesses a tranquillizing effect, memory retention improvement [83], cholinesterase inhibitory activity [84], anticonvulsant, CNS depressant, and antiepileptic [83] activities. *P. vulgare* showed neuro-psycho-pharmacological activity. Hence, the significant reduction of neuropsychological symptoms such as tension, anxiety, irritability, depression, mood swings [91], sleep changes [92–94], forgetfulness, and confusion could be justified because of the aforementioned properties of polypody rhizome. Female was able to significantly reduce premenstrual sleep disturbance [95] and irritability in PMS women. The authors reported that it could have central effects, perhaps by altering the serotonergic mechanism that regulates sleep [73].

Micronutrients improve quality of life, and nutrients have been used in the PMS symptoms alleviation for decades, but studies into their efficacy are rare. Research has documented the optimistic effects of micronutrients to treat various mental health concerns from stress to insomnia [56]. Furthermore, nutrition and dietary supplements are not only important in premenstrual syndrome and chronic diseases including diabetes and cancer [96]. Currently, ACOG has only recommended calcium (for the diminution of both mood symptoms and physical discomfort), and magnesium (to reduce breast tenderness, mood symptoms, and water retention) only supplements for PMS symptoms. Recent research showed evidence of the efficacy of herbs or dietary supplementation with other vitamins such as A, C, E, B6, and magnesium, as they might modify the hormonal or oxidative stress or anti-inflammatory profile of women experiencing PMS. Some studies support the relation between vitamin intake and symptom reduction in PMS. A study has shown the optimistic effect of calcium and vitamin E in PMS symptoms [97]. Retallick-Brown et al. [56] reported that vitamin B6 is used as an effective management for PM. They also suggested that nutritional treatment through micronutrients is also beneficial in PMS. In addition, they recommended that multiple micronutrients perhaps have better usefulness than vitamin B6 with severe symptoms. Initial evidence proposes that omega-3 has a useful action on the premenstrual physical and psychological symptoms. Omega-3 controls inflammation and tissue homeostasis [98].

A study reported that serum brain-derived neurotrophic factor (BDNF) levels in the luteal phase were lower in women with PMS. Zinc supplement administration in PMS caused a significant surge in BDNF than placebo. Zinc has antioxidant, anti-inflammatory, and antidepressant activities. This function is probably because of its contribution to increasing BDNF gene expression. It might influence inflammatory markers, viz., high sensitivity C-reactive protein (hs-CRP), and, thus, improve PMS symptoms [52]. Additionally, Zn^2+^ has an inhibitory role at neurosteroid sensitive extra synaptic GABA-A receptors [99]. A study found that in the hippocampus, zinc increases the BDNF protein and mRNA. It induces the matrix metalloproteinase (MMP) that triggers tropomyosin-related kinase proteinase, subsequently leading to pro-BDNF release from cells and converting to BDNF. BDNF supports the differentiation and survival of serotonin in neuron. In addition, zinc is thought to counteract the excess ROS in the body and inhibits NADPH oxidase [57].

The mechanism of how vitamin D decreases inflammation is based on findings that the action of 1, *α*, 25-dihydroxy vitamin D3 (1*α*,25 (OH)2D3) is mediated through Interleukins. Vitamin D3 inhibits IL-12 production in activated macrophages. However, no functional vitamin D response elements (VDREs) have been established in the genes IL-12A or IL-12B23. The initial effect of 1*α*,25 (OH)2D3 is a cyclical down regulation of IL-12B expression at the onset of inflammation, until sustaining the immune response with a possible level. Furthermore, a secondary effect of IL-10 occurs that turns off the IL-12B gene. In addition, IL-10 has an anti-inflammatory immune function and has effects on the brain and behavior, taking part in anxiety, modulation of mood, and depression symptoms. Thus, a study reported that the mean score of the total PMS symptoms significantly improved along with a significant improvement in inflammation marker. Based on their results, after treatment with vitamin D, increased serum levels of vitamin D ensued a noteworthy increase in serum TAC levels, imitating an enhancement in antioxidant status in PMS women [14].

Mostly, flowers and leaves are the most important parts of medicinal plants. The plant contains alpha-linolenic acid (ALA), delta6-fatty acryl desaturase, gamma-linolenic acid (GLA), pyrrolizidine alkaloids, delta8-sphingolipid desaturase, mucilage, potassium nitrate, calcium, resin, and mineral acids. *Echium amoenum* has anxiolytic, anti-inflammatory, antidepressant antianxiety, antioxidant, analgesic, and anti-obsessive-compulsive properties. Probably, the functional mechanism of *Echium Amoenum* (EA) depends on *γ* linolenic acid [55]. Flowers have GLA. GLA perhaps have anti-inflammatory and antioxidant properties. Various research studies have confirmed the beneficial effects of GLA on the severity and duration of PMS symptoms. Evening primrose oil is also one of the rich sources of GLA, and a review study approved its efficacy to decrease the severity of PMS symptoms after 4 to 6 months of treatment [100]. Indeed, the conversion of inflammation from a physiologic to a pathologic state can intensify PMS symptoms among individuals with PMS [79, 101, 102]. Additionally, PMS perhaps causes increased oxidative stress and decrease antioxidant capacity [103]. Furthermore, the most common anthocyanin, cyanidin 3-glucoside present in the petals of EA, has had neuroprotective effects and has customarily been used as an antidepressant and anxiolytic medicine in Asia [53].

Arabnezhad et al. [51] reported that curcumin improves serum vitamin D levels. Xin et al. [104] described that curcumin induces overexpression of vitamin D receptor (VDR) in osteoblasts and femurs probably involved in the protective effect of curcumin on bone loss created by microgravity although curcumin administration has no considerable effect on serum 1,25-(OH)2D3 concentration. VDR has an important effect on calcium absorption, bone mineralization rate, and regeneration, as it is a nuclear transcription factor that can modulate the activity of 1,25-(OH)2 [105]. VDR is also expressed in the ovarian tissue, placenta, endometrium, and fallopian tube epithelial cells. The VDR target genes such as CYP24, cytochrome P450 (CYP) 3A4, and TRPV6 can be stimulated and directly bound to curcumin and get triggered [106]. The role of vitamin D regarding the etiopathology of PMS and dysmenorrhea and its augmentation by curcumin might signify a reasonable mechanism for the useful therapeutic effect of curcumin in menstrual-associated symptoms. In addition, curcumin decreases gene expression of pro-inflammatory cytokines and suppresses nuclear factor kappa B (NF*κ*B) induction and high mobility group box 1 (HMGB1), cyclooxygenase-2, downregulation of ICAM-1 and MCP-1, procollagen type I, CD11b, and tissue inhibitor of metalloprotease-1 and induction of peroxisome proliferator-activated receptor-gamma (PPAR-*γ*) causing the amelioration of the development and enhancement of inflammation [107]. Furthermore, the antioxidant activity of curcumin is interrelated with triggering many antioxidant enzyme activities such as catalase, glutathione transferase, and heme-oxygenase-1 [108].

The polypody rhizome showed a significant reduction in PMS symptoms as it contains calcium and magnesium. Iron also plays a role in the metabolism of GABA and serotonin and is an essential cofactor for the tryptophan hydroxylase. The iron deficiency symptoms include cognitive problems, depression, and physical activity disorders. Few studies have reported a reduction in PMS symptom association after receiving iron. Polypody rhizome also contains iron, hence able to reduce PMS symptoms, similarly, has been reported in the wheat germ study [63].

Curcumin has anti-inflammatory, antioxidant, and neuroprotective activities. During the past decade, various scientific studies established that curcumin can curb the levels of neurotransmitters (dopamine, BDNF, norepinephrine, and serotonin) that are responsible for mood and behavior regulation [64, 109]. In stressed animals, curcumin prevented a diminution in hippocampal BDNF levels to levels comparable to imipramine. Furthermore, its treatment exerts strong antidepressant effects that are similar to the SSRIs (fluoxetine and imipramine), well-known antidepressant drugs [64]. Various physiological processes in the brain such as emotions, sleeping, attention, dreaming, and learning are controlled by norepinephrine. In an animal trial, curcumin increases dopamine levels in rodents and even noticeably decreases the effects of agents that induce reductions in the concentration of dopamine and adrenaline in the brain. In addition, the cyclooxygenase-2 enzyme (COX-2) produces prostaglandin E2, recognized to contribute a significant role in the premenstrual symptom's pathophysiology. It can downregulate the gene expression of the COX-2 enzyme and thus inhibits prostaglandin synthesis as per experimental research in animals. Khayat et al. [61] reported that levels of neurotransmitters were augmented by curcumin that improved mood and behavioral symptoms of PMS and through inhibition of COX-2 enzyme reduced physical symptoms of menstruation.

Chamomile tea is useful to reduce the severity of premenstrual symptoms as possessing anti-inflammatory, antioxidant, antianxiety, antihistamine, antispasmodic, analgesic, and antidepressant activities. Apigenin compound present in chamomile tea reduces the impact of hormones on the body and mind and excitation neurotransmitters, thereby soothing the hyperactive sympathetic nervous system. It modulates dopamine and serotonin activity and reduces the impact of depressive symptoms. Additionally, it reduces pain sensation by inhibiting the COX enzyme and reducing the inflammatory response of the immune system. Furthermore, its essential oil has antispasmodic and relaxing activities and is beneficial to calm the symptoms associated with PMS. A study compared the efficacy of the well-known anti-inflammatory medications, mefenamic acid, and ibuprofen with chamomile extract for relieving PMS. It is also effective for stress and anxiety relief as apigenin, Luteolin, glycine, and flavonoid since CNS stimulating molecule is a nerve relaxant. It is considered generally safe [8].

*N. sativa* has antioxidant, immunomodulatory, anti-inflammatory, and anticancer activities. Fresh extracted oil of. N. sativa contains more thymoquinone that reduced IL-6 levels significantly, whereas stored extracted oil inhibits IL-1beta and had a higher antioxidant activity. Thymoquinone reduces IL-6 levels significantly [110]. Fennel significantly reduces calcium influx recovery time and suppresses MAPK's phosphorylation as well as JNK, ERK, and p38 phosphorylation, with downstream signaling leading to degranulation and activation of respiratory bursts and in human neutrophils. Fascinatingly, fennel inhibiting protein kinase (PKA) inhibitor H89 partly restores the superoxide anion generation, along with the inhibitory effect on calcium influx recovery time, and indicates a role of the cAMP/PKA pathway in the anti-inflammatory effects of fennel. Fennel estragole exhibited anti-inflammatory effects *in vitro* by suppressing Nrf-2 and NF-lB pathways and *in vivo* (30 mg/kg) reduction of paw edema and leukocyte emigration in the peritoneal fluid [111]. The mechanism of action of *Allium sativum* is immuno-regulation and modulation of secretion of cytokines with therapeutic effects for metabolic syndrome such as antihypertensive, antidiabetic, and hypolipidemic properties. Persuasive evidence confirms the ability of garlic extract (AGE) to protect against oxidant-induced diseases, i.e., reduced risk of cancer, cardiovascular (CVD) disease, ageing, and stroke. In addition, it is effective in neurodegenerative disorders caused by oxidant-mediated brain cell damage, particularly Alzheimer's disease (AD) [52]. Several studies reported that garlic intake probably inhibits *β*-amyloid protein (A*β*) aggregation in the human brain. An experimental study in ovariectomized rats showed noteworthy antidepressant-like properties of a diet supplement containing garlic and black sesame [112]. Garlic, perhaps by reducing brain oxidative stress, reduces anxiety and depression behaviors in diabetic rats [113]. AGE possibly improves memory by affecting the glutamatergic, cholinergic, and GABAergic systems regarding cognitive impairment in A*β*-induced rats. An experiment in mice showed that garlic extract inhibits monoaminoxidase-A and monoaminoxidase-B, proving its antidepressant-like activity [114]. Research also showed that the administration of garlic in rats increases brain serotonin (5-hydroxytryptamine) levels [52]. Studies have shown that saffron has antioxidant properties. The phytoconstituent crocetin has stronger antioxidant activity than safranal, and the potential of crocetin was equivalent to that of Trolox and butyl hydroxyl toluene (BHT) [115].

**Figure 7:**
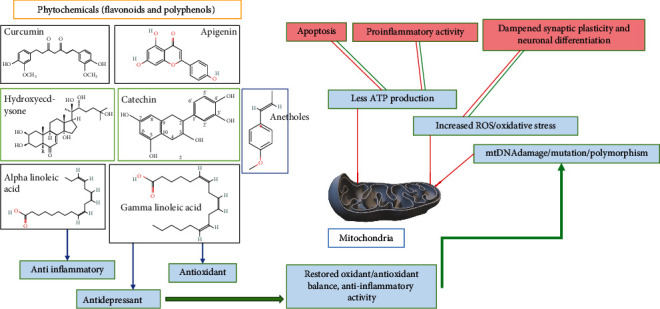
Antioxidant and anti-inflammatory effects of phytoconstituents in herbal medicine and nutritional supplements.

The mechanisms of action for phytoestrogen effects are unclear in PMS; however, there is indicative evidence that phytoestrogens may act through an estrogen receptor-independent. Various studies confirmed that phytoestrogens bind to estrogen receptors and show significant estrogenic-like effects [116]. The mechanism of anise is comparable to the selective moderators of estrogenic receptors that have agonistic effects as well as antagonistic effects on the estrogenic receptors. Anethole present in anise is considered the active estrogenic agent, and these herbs are considered phytoestrogens [53]. *N. sativa* may probably act directly as well as indirectly on the estrogen receptors and lead to substantial changes in the level of estrogen. Its estrogenic activity may perhaps be accredited to the presence of unsaturated fatty acids, which are established to possess estrogenic effects in animals, man, and cell cultures (Figure 7) [18, 116].

### 3.3. Does Computational Intelligence Have a Role in PMS Data Analysis for Future Modulation of Premenstrual Symptoms?

For example, we have applied computational intelligence techniques such as RF and SVM-radial basis function (SVM-RBF) to the experimental and placebo group. This study would be helpful for researchers, scientists, and doctors to understand the computational intelligence in this area.

In the experimental group, polypody dried rhizome, 2000 mg fine powdered, was administered in two capsules of 1 gm per oral twice a day from day 16 of the menstrual cycle to day 5 of the next cycle for three consecutive cycles. In the placebo group, edible cellulose powder (placebo), 2000 mg, was administered in two capsules of 1 gm per oral twice a day from day 16 of the menstrual cycle to day 5 of the next cycle for three consecutive cycles. The primary outcomes were changes in the total and subtotal score of PMSS for the occurrence and severity of PMS symptoms, and change in duration was assessed by the premenstrual tracker sheet. The secondary outcome was a change in EQ-5D-5L HRQoL. We have added the methods and results of the computational intelligence as shown in the following sections below.

#### 3.3.1. Computational Intelligence Methods

- RF classifier: Ensemble techniques are used to anticipate the final results of RF. A decision node, a leaf node, and a root node are all present in each of the decision trees. The leaf node is the output of each decision tree, and a majority voting process determines the ultimate conclusion [117–119]. If we have attributes Θ of a vector x and the decision tree based on these attributes is h (x, Θ), then the RF classifier can be defined:

(1) $$\begin{array}{c} R_{F}=\left[h\;\left(x,\Theta_{l}\right)\right],l=1,2,\cdots ,l. \end{array}$$

(ii) SVM classifier: It is utilized in the classification and regression methods of data analysis. For data prediction, SVM creates a hyperplane in infinite-dimensional space. The hyperplane with the greatest distance to the nearest training point of the class achieves the maximum accuracy. SVM was initially established in 1963 by prominent scientists Alexey Ya Chervonenkis and Vladimir N Vapnik. After 29 years, Vapnik's team created SVM for nonlinear data using a kernel method to boost the hyperplanes' maximum margin [120]
(iii) Performance measures: We used SVM-RBF kernel and RF classifiers for the classification of control and Polypody in terms of accuracy, precision, sensitivity, specificity, and area under the curve (AUC) [25, 30–32, 35, 121] with leave-one-out and cross-validation (CV) 5-fold models:

(2) $$\begin{array}{c} \text{Accuracy}=\left(\frac{\left(TP+TN\right)}{\left(TP+TN+FP+FN\right)}\right), \end{array}$$

(3) $$\begin{array}{c} \text{Sensitivity}=\left(\frac{TP}{\left(FN+TP\right)}\right), \end{array}$$

(4) $$\begin{array}{c} \text{Specificity}=\left(\frac{TN}{\left(FP+TN\right)}\right), \end{array}$$

(5) $$\begin{array}{c} \text{precision}=\left(\frac{TP}{\left(TP+FP\right)}\right), \end{array}$$

where TP is true positive, FP is false positive, TN is true negative, and FN is false negative.

#### 3.3.2. Computational Intelligence Results

**Figure 8:**
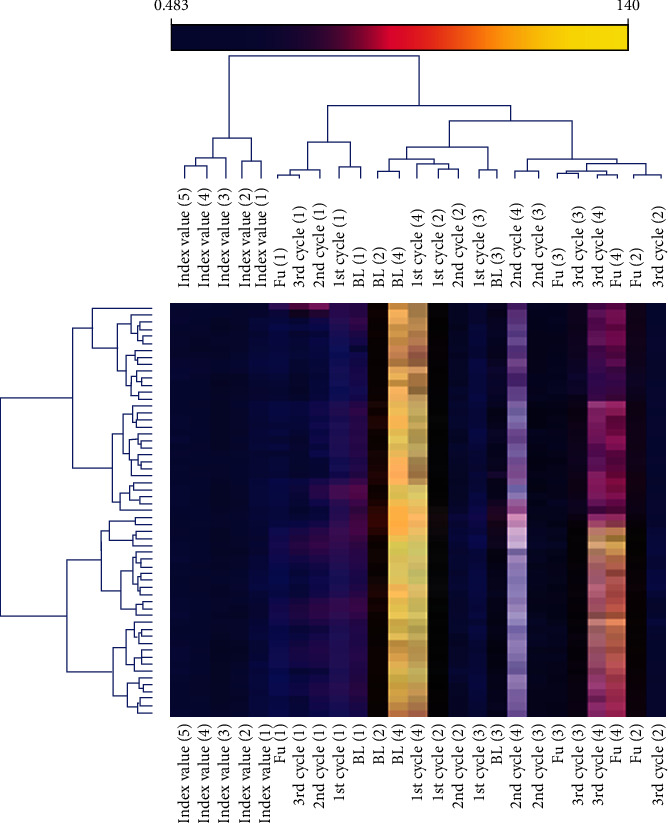
Heat map of the experimental and placebo group of the PMS. It showed the relation between premenstrual scale score, health-related quality of life index value related to the premenstrual psychosomatic and its behavioral symptoms of baseline (BL), three cycles with treatments, and one follow-up (FU) without treatment.

We have designed the heat map of the experimental data related to the premenstrual syndrome mentioned in Figure 8. It showed the relationship between control and polypody experimental data. The leave-one-one-out classification model of the RF classifier achieved maximum accuracy in terms of accuracy (90%), recall (90%), specificity (90%), and precision (90.20%). Moreover, the CV-5 classification model of the RF classifier achieved maximum accuracy in terms of accuracy (88.30%), recall (88.30%), specificity (88.30%), and precision (88.40%).

However, our RF leave-one-one-out models are more suitable for the classification of the experimental data related to premenstrual psychosomatic and its behavioral symptoms shown in Table 4. Furthermore, the random forest classifier achieved maximum accuracy in terms of accuracy, recall, specificity, and precision.

## 4. Discussion

In this review, comprehensively, we highlighted the low risk and high quality of 25 RCTs, etiopathogenesis, the contribution of oxidative stress, inflammation leading to mitochondrial changes, and the role of phytochemical constituents in premenstrual psychosomatic and its behavioral symptoms. Additionally, as per our knowledge, it is the first time that computational intelligence technique has been applied to detect premenstrual psychosomatic and its behavioral symptoms assessed by PMSS questionnaire in women who used Polypodium *vulgare* L. herb vs placebo towards future modulation of computational intelligence in clinical trials.

This study covered the required details for the up-to-date research work on premenstrual psychosomatic and its behavioral symptoms. We found that in previous work various tools were used to assess PMS symptoms. We reviewed twenty-five articles with 1949 participants (mean ± SD: 77.96 ± 22.753) using PRISMA techniques. Besides, our research questions covered mitochondrial changes, oxidative stress and antioxidants, inflammation, anti-inflammatory herbal medicine, and nutritional supplements in PMS. In addition, bioactive molecules and pharmacological activities of herbs effective in PMS symptoms and the mechanism of action have been highlighted.

**Figure 9:**
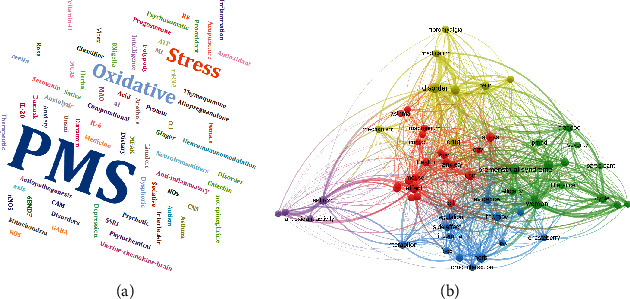
Closest terms of the present study based on (a) word cloud and (b) network visualization.

We also designed the word cloud and network visualization of the current review mentioned in Figure 9 [39, 40, 122, 123]. A word cloud is advantageous to cover all valuable keywords of the present study in a single diagram. Additionally, we have designed a brief description of the proposed RQs based on MeSH terms using network visualization techniques [123], shown in Table 5. This technique is valuable to get the exact MeSH keywords related to a particular disease, and it is a new tool to analyze many previously published data. Additionally, manually, it is tough to determine the closest terms for any big database. These tools will upwhirl a new way to envisage the dataset through software. It easily divides the previously published articles based on terms in the cluster. For better comprehension, previous studies on the role of oxidative and inflammation in premenstrual symptoms, nutritional supplements, and herbal medicine were retrieved from Scopus, PubMed, and PROSPERO databases were retrieved.

Previously, the researchers reviewed inflammation, oxidative stress in PMS, various nutritional supplements, and herbal medicines in PMS. In [1], a prospective cohort study examined the serum antioxidant vitamin concentrations and oxidative stress markers in PMS. Granda et al. determined oxidative stress, inflammation markers, and antioxidant status in PMS women. They used to update the data until January 2021 from PubMed and Scopus to cover the literature of the study. Koohpayeh et al. [124] investigated the effects of *Rosa damascena* on menstruation-related pain, anxiety, fatigue, headache, and bloating. In [8], the authors investigated chamomile for the treatment of PMS syndrome and collected literature reviews from various databases from 1990 to 2019. They included eight studies. Verkaik et al. [125] investigated for *Vitex agnus castus*, collected data up to January 2016 from various databases, and included 17 studies. Csupor et al. [126] collected data from three clinical trials from 21 clinical trials to compare the efficacy of Ze 440 and BNO 1095 with placebo for the management of PMS. The effectiveness and safety of Iranian herbal medicines for PMS investigators collected data up to 2017 and included 18 RCTs [127]; it included 10 RCTs from 17 RCTs for herbal treatment for PMS [20]. Canning et al. [71] investigated dietary supplements and herbal medicines for PMS. Tu et al. [128] recommended that education and communication with parents might reduce parental stress and anxiety. Lai group designed a comprehensive survey on insomnia and bruxism sleep disorders based on PRISMA, RQs, and network visualization techniques. As per our knowledge, researcher used these methods on the PMS, oxidative stress, and inflammation with world clouds and computational intelligence techniques.

However, our study is different from others regarding details related to etiopathogenesis, the contribution of oxidative stress and inflammation leading to mitochondrial changes in premenstrual symptoms, the therapeutic implication of herbal medicines and nutritional supplements, and the mechanisms of action of these herbal medicines and nutritional supplements. Additionally, we applied computational intelligence techniques to the experimental and placebo groups. These techniques would be helpful for the detection, treatment, and localization-related to our studies. Furthermore, network visualization for retrieval of a literature review from the Scopus database was also performed. This study will benefit academicians, researchers, and scientists to work in this area and use artificial intelligence for data retrieval from various databases. In addition, our closest terms would be helpful for the researchers, scientists, students, doctors, and academicians to find the closest article related to our study.

## 5. Research Gap and Future Recommendations

The prerequisite of additional information that replicates the effect of premenstrual psychosomatic and its behavioral symptoms to appropriately reply to the self-reported questionnaire and questions on instinct decisions on the physical and mental health of an individual with specific criteria would give a further and definite reason for PMS. The measurement limitation might be present in those questions that are inclusive of the self-reported questionnaire as there could be personal questions that some participants may find challenging to answer. In this paper, that gap is apparent and thus would need further exploration. More research, including a larger sample of subjects, would allow evidence to support a relationship between oxidative stress, inflammation, and PMS and the therapeutic implication of herbal medicine and nutritional supplements in premenstrual psychosomatic and its behavioral symptoms.

In literature research, oxidative stress and inflammation are still not very comprehensively discussed, so what is recommended is more in-depth qualitative and quantitative studies with more significant sample sizes. Indeed, further investigation is essential to explore the relationship between a given mechanism of action of herbal medicine and nutritional supplements in human trials, although few animal studies have proven anti-inflammatory and antioxidant properties of herbal medicine and nutritional supplements beneficial in premenstrual psychosomatic and its behavioral symptoms. More analysis is needed at the molecular and cellular level to further guide. Future studies must be started to better identify possible mitochondrial dysfunction linked to PMS and the molecular mechanisms and software development for the automatic detection related to mitochondrial dysfunction targeting the cognitive, psychomotor, and affective domains in PMS. Researchers would develop more specific scales to define PMS and causal connections between inflammatory and marker markers. Emerging technologies like machine learning, brain network, quantum techniques, computer vision, and block-chain technology help to analyze experimental RCTs data.

## 6. Conclusion

This review can be a milestone towards experimenting with herbal medicines and nutritional supplements that are potentially effective natural alternatives to relieve PMS symptoms as they possess bioactive molecules such as curcumin, allicin, anethole, thymoquinone, cyanidin 3-glucoside, pyrrolizidine alkaloids, omega fatty acid, polypodin A and polypodin B, sesquiterpene, jatamansone, ursolic and oleanolic acids, magnesium, zinc, calcium, beta-carotene, linoleic acid, and gamma linoleic acid not only have antioxidants and anti-inflammatory properties but also other various activities (GABAA receptor agonist, MAO inhibitors, serotonergic, antidepressant, sedative, and analgesic). Further, computational intelligence analyses of the experimental data of one of the RCTs using computational intelligence techniques showed the accuracy of the data. Hence, it is recommended that machine learning techniques have potential and are useful in clinical trials. However, more rigorous research studies are recommended for in-depth knowledge of the efficacy of bioactive molecules on premenstrual syndrome in clinical trials.

## Figures and Tables

**Table 1:** Various inclusion and exclusion criteria.

Criteria	Inclusion	Exclusion	Justification
Subject	Patients with premenstrual symptoms	No exclusion	Included all PMSS scales
Language	English and Persian	Other languages	Probabilities of misapprehension of data in other languages may therefore disturb the accuracy
Access	Can access full text	Cannot access full text	To confirm the explanation of an article more precisely
Methodology	All	No exclusions	All methodologies were included for a holistic view
Nature of the article	Research and Survey	Observational studies, review, book, and editorial	Review articles comprehending a generalized discussion over a topic would not sufficiently answer our specific research question

**Table 2:** The characteristics of the published RCTs on herbal medicines and nutritional supplements and their bioactive molecules with pharmacological activities.

Blinding in RCT	Part.	Age (years)	Tools	Exp. group	Cont. Group	Duration of Rx	Result	Adv. Event	Bioactive molecules	Pharm. Actions	Ref.
Triple-blind	76	18-24	PSST, VAS score <8 for dysmenorrhea	One capsule (500 mg of curcuminoid+5 mg piperine) (n =38)	Placebo (*n* = 38)	Daily for 7days before until 3days after menstruation for 3 menstrual cycles	Curcumin significantly increased the median serum levels of vitamin D, liver function enzyme test, but did not affect blood glucose	Not reported	Curcumin	Antioxidant, anti-inflammatory, antimicrobial and anticarcinogenic	[51]
Double-blind	129	15-49	PSST	400 mg (1.1 mg allicin) (*n* = 64)	Placebo (*n* = 65)	One tablet daily for 3 cycles	Significant reduction in the symptoms	Reported	Allicin, inhibits MAO enzyme, acts as an antidepressant hyperforin act as SRI	Anti-depressant, anti-inflammatory antioxidant, immunomodulator	[52]
Double-blind	84	18-35	PSST	110 mg capsules of anise (*n* = 42)	Placebo (starch) (*n* = 42)	TID, 7 days before the start of the menstruation and first 3 days during menses for 2 consecutive menstruations	Decreased the symptoms in comparison to placebo	Reported	Anethole	Antioxidant, hypoglycemic, hypolipidemic, anticonvulsant selective moderator of estrogenic receptors	[53]
Double-blind	84	18-35	DASS 21, COPE	Oral capsules containing 500 mg of *Nigella sativa* seeds (*n* = 42)	Placebo (*n* = 42)	Same as above	Significant reduction in overall severity of premenstrual syndrome in the intervention group	Not reported	Thymoquinone	Increase brain GABA, anti-anxiety, antioxidant, anti-inflammatory	[54]
Double-blind	84	20-35	PSST	450 mg capsules of flowers of *Echium amoenum* (*n* = 42)	Placebo (*n* = 42)	TID from the 21st day to the 3rd day of their next cycle for 2 consecutive cycles	More effectively symptoms were improved in experimental group	Reported	Cyanidin 3-glucoside, (anthocyanin) GLA, ALA, pyrrolizidine alkaloids, and calcium and mineral acids	Anti-inflammatory, antioxidant, analgesic, anxiolytic, sedative and anticonvulsant	[55]
Double-blind	72	>18	DRSP DASS-42	80 mg B6 (*n* = 35)	EM Power Plus (*n* = 35)	Four capsules BID from day 1 of menstruation of new cycle till 2 months	Both groups were effective in PMDD	Not reported	Omega fatty acid	Antioxidant	[56]
Double-blind	60	18-30	30-item questionnaire based on DSM-VI	30-mg zinc gluconate (*n* = 30)	Placebo (*n* = 30)	12 weeks	Significant reductions in symptoms and increase in BDNF factor and total antioxidant capacity in experimental group	Not reported	Increases in BDNF Expression, inhibits GABA A receptor	Antidepressant, anti-inflammatory, antioxidant	[57]
Single-blind	60	18-45	PMSS scale HRQoL: EQ-5D-5L	1000 mg of *P. vulgare*. (*n* = 30)	Placebo (*n* = 30)	2 capsules, BID from day 16 of the menstrual cycle to day 5 of the next cycle for 3 consecutive cycles	Significant decrease in symptoms and improvement in EQ-5D-5L index value in the experimental group	Reported	Polypodin A and polypodin B bioflavonoid, tannin, phenols	Stress modulator, antidepressant, antioxidant, immunomodulator, analgesic, neuroprotective, anti-inflammatory, antioxidant properties, effects on the rennin-angiotensin system, and increase 5- hydroxytryptamine in the brain	[58]
Double blind	44	18-25	BDI-S, BAI	Vitamin D3 (50,000 IU) (*n* = 22)	Placebo pearl (*n* = 22)	Every 15 days for 4 months	25(OH)D, serum IL-12 and TAC levels improved significantly in the test group	Not reported		Antidepressant, anti-inflammatory, antioxidant	[14]
Single-blind	101	20-50	PMTS-O, VAS	Pollen pistil extract serelys The tablets administered as the active treatment contained 120 mg PI 82 and 40 mg GC FEM. 2 serelys tablets (320 mg per day) (*n* = 50)	Placebo (n =51)	4 months	Significant symptom reduction with serelys treatment	Not reported	Flavonoids, tannins and polyphenols		[59]
Single-blind	60	18-45	PMTS-O, PMTS-SR, VAS score	666 mg of *N. jatamansi* (*n* = 30)	Placebo (*n* = 30)	Orally, BID for the 15 days prior to the expected date of menses for 2 cycles	Significant reductions in the test group than the placebo	Not reported	Sesquiterpene jatamansone Inhibit GABA and MAO	Anti-depressant neuroprotective activity. Preventing cognitive impairment and neurodegeneration	[60]
Double blind	70	Premenopausal women	DSM-IV	Curcumin (100 mg) (*n* = 42)	Placebo (brown sugar) (*n* = 35)	2 capsules BID daily for 7 days before and three days during menstruation for three successive cycles	Significant reduction in severity of PMS symptoms	Not reported	Curcumin	Antidepressant due to serotonergic system, anti-inflammatory, antioxidant, neuroprotective	[61]
Double-blind	100	University students	GHQ-28, PSST	*M. officinalis* essence (1200 mg) (*n* = 50)	Placebo (*n* = 50)	Daily for three cycles from day 1 of last day of menstrual cycle	Significant reduction of somatic, psychological and social symptoms	Not reported	Triterpenoids (ursolic and oleanolic acids) accountable for the inhibition of rat brain GABA transaminase.	Anxiolytic, anti-depressant spasmolytic, sedative, antioxidant, immunomodulatory antiviral and antispasmodic	[62]
Triple-blind	100	20-45	DSR, BDI	Wheat germ extract (400 mg capsules)	Placebo (*n* = 50)	TID a day between the 16th day of the menstrual cycle to the 5th day of the next menstrual period for two cycles	Significant reduction in symptoms	Not reported	Magnesium, zinc, calcium, antioxidants including beta-carotene (for vitamin A), thiamin, folic acid, vit E, C, B12, B6, riboflavin, niacin, iron, amino acids, and enzymes. Linoleic acid	Antioxidant, anti-inflammatory	[63]
Double-blind	70	Students	DSM-IV Fasting serum BDNF level	100 mg/12 h. curcumin capsules (*n* = 35)	Placebo (*n* = 35)	3 successive menstrual cycles and each cycle ran 10 days (in each menstrual cycle 7 days before and 3 days after onset of menstrual bleeding)	Significantly increase in BDNF levels and PMS symptoms were significantly reduced in the test than placebo group	Not reported	Curcumin	Modulating level of BDNF, anti-depressant	[64]
Triple-blind	80	18-30	DSM IV-TR	1500 mg primrose oil (*Oenothera biennis*) (*n* = 40)	Placebo (*n* = 40)	TID for three months	Reduction of symptom severity in both primrose and placebo group	Not reported	Linoleic acid and gamma linoleic acid	Antioxidant, anti-inflammatory	[65]
Double-blind	70	18-35	DSM-IV	Two ginger capsules 250 mg (*n* = 35)	Placebo (*n* = 35)	BID (7days) before menstruation to three days after menstruation for three cycles	Reduction in symptoms	Reported	Sesquiterpenoids, with (-)-zingiberene. Sesquiterpene lactones (SLs) are responsible for their anti-inflammatory activity	Immuno-modulatory, anti-tumorigenic, anti-inflammatory, anti-apoptotic, anti-hyperglycemic, anti-lipidemic, and anti-emetic actions (Rehman et al. 2011)	[66]
Single-blind	60	13-40	PMTS-SR, PMTS-O	*Vitex agnus castus* seed 1 gm and *Mentha piperita* distillate (Arq Pudina) 36 ml (*n* = 30)	Placebo (*n* = 30)	BID daily, 10 days before menstruation in every cycle for 3 cycles	A significant reduction in scores was observed in the test group than placebo (*p* < 0.01)	Not reported	Flavonoid casticin phenols, tannin, *α*-pinene, limonene, *β*-caryophyllene, sabinene, and *β*-farnesene	Anxiolytic, antioxidant, chemopreventive, immunomodulatory and cytotoxicity, antimicrobial, antifungal, antinociceptive, opioidergic, antiepileptic neuroprotective	[67]
Double-blind	128	Child bearing age	DSR	*Vitex agnus* (40 drops of in a glass of fruit juice) (*n* = 62)	Placebo (*n* = 66)	Orally in the morning from the 6th day prior menses until menstruation, for 6 consecutive cycles	Significant relief of mild and moderate PMS symptoms	Not reported	Flavonoid casticin phenols, tannin, *α*-pinene, limonene, *β*-caryophyllene, sabinene, and *β*-farnesene	Antioxidant, chemo preventive, immunomodulatory and cytotoxicity, antimicrobial, antifungal, antinociceptive, opioidergic, antiepileptic neuroprotective	[68]
Double blind	80	18-30	DSM-IV	10 drops of citrus essence (*n* = 40)	Placebo (*n* = 40)	TID during the luteal phase for two cycles	Reduction in the severity of premenstrual syndrome in the experimental group	Not reported	*β*-Pinene, limonene, terpinolene, *α*- and *γ*-terpinene, 1,8-cineol, *α*-pinene	Enhancing mood and effects sedation, antispasmodic, anti-inflammatory, antioxidant (Hsouna 2017)	[69]
Double blind	60	—	DRSP-Q	30 drops of fennel extract (*n* = 30)	Placebo (*n* = 30)	Every 8 hours for 3 days during menses for three months	Significantly greater improvement with the fennel extract than placebo	Reported	Anethole, estragole	Prostaglandin inhibitor, antioxidant, anti-inflammatory, analgesic (Korinek et al., 2021)	[70]
Double-blind	36	18-45	DSR	*Hypericum perforatum* 450 mg (*n* = 19)	Placebo (*n* = 17)	BID for cycle 4-10 months	Significantly benefit on symptoms	Reported	Hypericin inhibits MAO enzyme and acts as an antidepressant Hyperforin acts as SRI	Neuroprotective, anti-depressant, antiangiogenic Anti-inflammatory wound healing and anti-nociceptive effect. Antioxidant	[71]
Single-blind	90	18-30	DSM-IV, BDI	*G. biloba* L. tablets (containing 40 mg leaf extracts) (*n* = 45)	Placebo (*n* = 45)	TID from the 16th day of the menstrual cycle to the 5th day of the next cycle for 2 cycles	Significant decrease in the overall severity of symptoms in the experiment group	Reported	Quercetin is an effective inhibitor of histamine release Bioflavonoids are stress modulator	Anti-inflammatory, anxiolytic, anti-depression	[13]
Double blind	50	20-45	Premenstrual daily symptoms score	Capsule saffron 30 mg/day (*n* = 25)	Capsule placebo (*n* = 25)	15 mg BID for a two menstrual cycles	Significant difference in the total PMS and HDRS scores	Reported	Crocin and safranal of saffron inhibit the reuptake of dopamine, norepinephrine and serotonin	Antioxidant, anti-depressant, antispasmodic, anticancer agent and memory enhancer	[72]
Double blind	101	20-50	Steiner premenstrual tension syndrome and VAS	Femal, 160 mg (*n* = 50)	Placebo (*n* = 51)	BID for four menstrual cycles	Significant reduction in PSF and PMTS scale	Reported	Thymol, camphor	Serotonin reuptake inhibitors	[73]

Total participants: 1949 with mean ± SD: 77.96 ± 22.753, variance: 517.71; and CI: 4.55. BAI: Beck Anxiety Inventory, BDI: Beck Depression Inventory; BDI-SL, Beck Depression Short Inventory; COPE: calendar of premenstrual experience; DSR: daily symptom report; DRSP-Q: daily record of severity of problem questionnaire; daily premenstrual syndrome questionnaire with DSM-IV; HDRS: Hamilton Depression Rating Scale: PMTS-O: Steiner premenstrual tension observer questionnaire; PMTS-SR: Steiner premenstrual tension self-rating questionnaire; PSST: Premenstrual Symptoms Screening Tool questionnaire; SD: standard deviation; CI: confidence interval.

**Table 3:** Randomized controlled studies showing risk of bias.

Study	Randomization	Approach of randomization appropriate	Blinding	Approach of blinding appropriate	Presentation of withdrawals and dropouts	Presentation of the inclusion/exclusion criteria	Adverse effects described	Statistical analysis described	Modified Jadad scale
Ozgoli et al., 2009 [13]	1	1	0.5	1	1	1	1	1	7.5
Heidari et al., 2019 [14]	1	1	1	1	1	1	0	1	7
Arabnezhad et al., 2022 [51]	1	1	1	1	1	1	0	1	7
Jafari et al., 2021 [52]	1	1	1	1	1	1	1	1	8
Farahmand et al., 2020 [53]	1	1	1	1	1	1	1	1	8
Maskani et al., 2020 [54]	1	1	1	1	1	1	0	1	7
Farahmand et al. 2020 [55]	1	1	1	1	1	1	1	1	8
Brown et al., 2020 [56]	1	1	1	1	1	1	0	1	7
Jafari et al., 2020 [57]	1	1	1	1	1	1	0	1	7
Khanam and Sultana, 2020 [58]	1	1	0.5	1	1	1	1	1	7.5
Winther et al., 2018 [59]	1	1	1	1	1	1	0	1	7
Malik et al., 2018 [60]	1	1	0.5	1	1	1	0	1	6.5
Khayat et al., 2015 [61]	1	1	1	1	1	1	0	1	7
Akbarzadeh et al., 2015 [62]	1	0	1	1	0	1	0	1	5
Ataollahi et al. 2015 [63]	1	1	1	0	0	1	0	1	5
Fanaei et al., 2016 [64]	1	1	1	1	1	1	0	1	7
Saki et al., 2015 [65]	1	1	1	0	0	1	0	1	5
Khayat et al., 2014 [66]	1	0	1	1	1	1	1	1	7
Shameem et al., 2014 [67]	1	1	0.5	1	0	1	0	1	5.5
Zamani et al., 2012 [68]	1	1	1	1	0	1	0	1	6
Ozgoli et al., 2011 [69]	1	1	1	1	1	1	0	1	7
Delaram et al., 2011 [70]	1	0	1	0	0	1	1	1	5
Canning et al., 2010 [71]	1	1	1	1	1	1	1	1	8
Hosseini et al., 2008 [72]	1	1	1	1	1	1	1	1	8
Gerhardsen et al., 2008 [73]	1	1	1	1	1	1	1	1	8

**Table 4:** Performance of the system is based on control and Polypody classification using computational intelligence techniques such as SVM-RBF and RF classifiers.

Classifier	Model	Accuracy (%)	Recall (%)	Specificity (%)	Precision (%)	AUC (%)
SVM-RBF	Leave one out	88.30	88.30	88.30	88.40	91.30
RF		**90.00**	**90.00**	**90.00**	**90.20**	89.10
SVM-RBF	CV-5	86.70	86.70	86.70	86.70	91.60
RF		88.30	88.30	88.30	88.40	89.30
Mean		88.325	88.325	88.425	90.325	88.325
±Standard deviation		1.166	1.166	1.237	1.132	1.166
Variance		1.361	1.361	1.531	1.281	1.361

**Table 5:** Description of the proposed research questions based on closest terms using the network visualization model.

S. No.	Research questions	Closest terms
1.	What is etiopathogenesis and the role of oxidative stress, inflammation leading to mitochondrial changes in premenstrual psychosomatic, and its behavioral symptoms?	Anxiety, depression, disorder, menstrual cycle, pain, patient, placebo, PMS, premenstrual symptoms, premenstrual syndrome, relationship, role, severity, symptom, behavioral, and woman
2.	What are the various mechanisms of action in nutritional supplements and herbal medicines with their phytochemical constituents in premenstrual psychosomatic and its behavioral symptoms?	Anxiety, antioxidants, biomarkers, calcium, clinical trials, depression, inflammation, infection, menstrual cycle, patients, premenstrual syndrome, roots, superoxide, symptom, woman, BDR, brain, herbal medicine, nutrition, phytochemicals, symptom, PMS, protein, ROS, and woman
3.	Does computational intelligence have a role in PMS data analysis for future modulation of premenstrual symptoms?	AI, analysis, data, classification, classifier, recognition, computational intelligence, machine learning, PMS, prediction, RF, and SVM

## Data Availability

The data used to support the findings of this study are included within the article.
